# Interhospital transfer vs. direct presentation of patients with a large vessel occlusion not eligible for IV thrombolysis

**DOI:** 10.1007/s00415-020-09812-5

**Published:** 2020-04-07

**Authors:** Laura C. C. van Meenen, Adrien E. Groot, Esmee Venema, Bart J. Emmer, Martin D. Smeekes, Geert Jan Kommer, Charles B. L. M. Majoie, Yvo B. W. E. M. Roos, Wouter J. Schonewille, Bob Roozenbeek, Jonathan M. Coutinho, the MR CLEAN Registry Investigators, Diederik W. J. Dippel, Aad van der Lugt, Charles B. L. M. Majoie, Robert J. van Oostenbrugge, Wim H. van Zwam, Jelis Boiten, Jan Albert Vos, Josje Brouwer, Sanne J. den Hartog, Wouter H. Hinsenveld, Manon Kappelhof, Kars C. J. Compagne, Robert-Jan B. Goldhoorn, Maxim J. H. L. Mulder, Ivo G. H. Jansen, Bob Roozenbeek, Adriaan C. G. M. van Es, Bart J. Emmer, Jonathan M. Coutinho, Wouter J. Schonewille, Marieke J. H. Wermer, Marianne A. A. van Walderveen, Julie Staals, Robert J. van Oostenbrugge, Wim H. van Zwam, Jeannette Hofmeijer, Jasper M. Martens, Geert J. Lycklamaà Nijeholt, Jelis Boiten, Sebastiaan F. de Bruijn, Lukas C. van Dijk, H. Bart van der Worp, Rob H. Lo, Ewoud J. van Dijk, Hieronymus D. Boogaarts, J. de Vries, Paul L. M. de Kort, Julia van Tuijl, Jo Jo P. Peluso, Puck Fransen, Jan S. P. van den Berg, Boudewijn A. A. M. van Hasselt, Leo A. M. Aerden, René J. Dallinga, Maarten Uyttenboogaart, Omid Eschgi, Reinoud P. H. Bokkers, Tobien H. C. M. L. Schreuder, Roel J. J. Heijboer, Koos Keizer, Lonneke S. F. Yo, Heleen M. den Hertog, Emiel J. C. Sturm, Paul Brouwers, Wim H. van Zwam, Geert J. Lycklamaà Nijeholt, Marianne A. A. van Walderveen, Marieke E. S. Sprengers, Sjoerd F. M. Jenniskens, René van den Berg, Albert J. Yoo, Ludo F. M. Beenen, Alida A. Postma, Stefan D. Roosendaal, Bas F. W. van der Kallen, Ido R. van den Wijngaard, Jasper M. Martens, Lonneke S. F. Yo, Joost Bot, Pieter-Jan van Doormaal, Anton Meijer, Elyas Ghariq, Reinoud P. H. Bokkers, Marc P. van Proosdij, G. Menno Krietemeijer, Jo P. Peluso, Hieronymus D. Boogaarts, Rob Lo, Dick Gerrits, Wouter Dinkelaar, Auke P. A. Appelman, Bas Hammer, Sjoert Pegge, Anouk van der Hoorn, Saman Vinke, Robert J. van Oostenbrugge, Wim H. van Zwam, Geert J. Lycklamaà Nijeholt, Jelis Boiten, Hester F. Lingsma, Naziha el Ghannouti, Martin Sterrenberg, Corina Puppels, Wilma Pellikaan, Rita Sprengers, Marjan Elfrink, Michelle Simons, Marjolein Vossers, Joke de Meris, Tamara Vermeulen, Annet Geerlings, Gina van Vemde, Tiny Simons, Cathelijn van Rijswijk, Gert Messchendorp, Nynke Nicolaij, Hester Bongenaar, Karin Bodde, Sandra Kleijn, Jasmijn Lodico, Hanneke Droste, Maureen Wollaert, Sabrina Verheesen, D. Jeurrissen, Erna Bos, Yvonne Drabbe, Michelle Sandiman, Nicoline Aaldering, Berber Zweedijk, Mostafa Khalilzada, Jocova Vervoort, Eva Ponjee, Sharon Romviel, Esmee Venema, Vicky Chalos, Ralph R. Geuskens, Tim van Straaten, Saliha Ergezen, Roger R. M. Harmsma, Daan Muijres, Anouk de Jong, Olvert A. Berkhemer, Anna M. M. Boers, J. Huguet, P. F. C. Groot, Marieke A. Mens, Katinka R. van Kranendonk, Kilian M. Treurniet, Manon L. Tolhuisen, Heitor Alves, Annick J. Weterings, Eleonora L. F. Kirkels, J. H. F. Voogd, Lieve M. Schupp, Sabine Collette, Adrien E. D. Groot, Natalie E. LeCouffe, Praneeta R. Konduri, Haryadi Prasetya, Nerea Arrarte-Terreros, Lucas A. Ramos

**Affiliations:** 1 Department of Neurology, Amsterdam UMC, University of Amsterdam, Amsterdam, The Netherlands; 2 Department of Neurology and Department of Public Health, Erasmus MC University Medical Center, Rotterdam, The Netherlands; 3 Department of Radiology and Nuclear Medicine, Amsterdam UMC, University of Amsterdam, Amsterdam, The Netherlands; 4 Emergency Medical Services North-Holland North, Alkmaar, The Netherlands; 5 National Institute of Public Health and the Environment, Center for Nutrition, Prevention and Health Services, Bilthoven, The Netherlands; 6 Department of Neurology, St. Antonius Ziekenhuis, Nieuwegein, the Netherlands; 7 Department of Radiology and Nuclear Medicine, Erasmus MC University Medical Center, Rotterdam, The Netherlands

**Keywords:** Patient transfer, Thrombectomy, Thrombolysis, Stroke

## Abstract

**Background and purpose:**

Direct presentation of patients with acute ischemic stroke to a comprehensive stroke center (CSC) reduces time to endovascular treatment (EVT), but may increase time to treatment for intravenous thrombolysis (IVT). This dilemma, however, is not applicable to patients who have a contraindication for IVT. We examined the effect of direct presentation to a CSC on outcomes after EVT in patients not eligible for IVT.

**Methods:**

We used data from the MR CLEAN Registry (2014–2017). We included patients who were not treated with IVT and compared patients directly presented to a CSC to patients transferred from a primary stroke center. Outcomes included treatment times and 90-day modified Rankin Scale scores (mRS) adjusted for potential confounders.

**Results:**

Of the 3637 patients, 680 (19%) did not receive IVT and were included in the analyses. Of these, 389 (57%) were directly presented to a CSC. The most common contraindications for IVT were anticoagulation use (49%) and presentation > 4.5 h after onset (26%). Directly presented patients had lower baseline NIHSS scores (median 16 vs. 17, *p* = 0.015), higher onset-to-first-door times (median 105 vs. 66 min, *p* < 0.001), lower first-door-to-groin times (median 93 vs. 150 min; adjusted *β* = − 51.6, 95% CI: − 64.0 to − 39.2) and lower onset-to-groin times (median 220 vs. 230 min; adjusted *β* = − 44.0, 95% CI: − 65.5 to − 22.4). The 90-day mRS score did not differ between groups (adjusted OR: 1.23, 95% CI: 0.73–2.08).

**Conclusions:**

In patients who were not eligible for IVT, treatment times for EVT were better for patients directly presented to a CSC, but without a statistically significant effect on clinical outcome.

**Electronic supplementary material:**

The online version of this article (10.1007/s00415-020-09812-5) contains supplementary material, which is available to authorized users.

## Background

Intravenous thrombolysis (IVT) is the standard treatment for patients with acute ischemic stroke (AIS) [1]. Patients with a large vessel occlusion (LVO) of the anterior circulation are additionally treated with endovascular treatment (EVT) [2]. In most countries, paramedics transport patients with a suspected AIS to the nearest primary stroke center (PSC) for diagnostic work-up and to initiate IVT. Patients who are eligible for EVT are subsequently transferred to a comprehensive stroke center (CSC). Studies show that this ‘drip-and-ship’ system delays initiation of EVT by 40–106 min and decreases the chance of a good clinical outcome by approximately 10% [3–5]. Despite this clear disadvantage, the ‘drip-and-ship’ system is currently the most feasible, because accurately diagnosing an LVO in the prehospital setting is challenging. Directly presenting all patients with suspected AIS to a CSC would overburden these hospitals. In addition, due to longer initial travel times, a centralized model could delay initiation of IVT, and thus negatively impact patient outcome in patients who are not eligible for EVT [5].

Approximately 20% of patients who undergo EVT in routine practice do not receive IVT because of a contraindication for alteplase [6]. Most of these contraindications, such as anticoagulation use and duration of symptoms > 4.5 h, can be easily determined in the ambulance. For patients with such a contraindication for IVT, no valuable time would be lost by bypassing the PSC and going directly to a CSC. In the current study, we analyzed workflow times and clinical outcomes after EVT in patients who were not eligible for IVT, and compared these outcomes between patients who were directly presented to a CSC to those initially presented to a PSC.

## Methods

Data will not be made available to other researchers, as no patient approval was obtained for sharing coded data. However, syntax and output files of statistical analyses may be made available on request.

### Study design and population

We used data from the MR CLEAN Registry. The MR CLEAN Registry is a nationwide, prospective cohort study, in which all patients who have undergone EVT for AIS in the Netherlands since completion of the MR CLEAN trial (March 2014) until December 2018 have been registered. Permission to carry out this study was granted by the medical ethics committee of the Erasmus University Medical Center in Rotterdam. Detailed methods of the MR CLEAN Registry have previously been reported [6]. For the current study, we used data collected from March 2014 until November 2017 (Registry part I and II). We included patients who had undergone EVT for AIS, and did not receive IVT. In-hospital strokes were excluded.

### Definitions and outcomes

EVT was defined as arterial puncture in the angiography suite, with the objective to perform mechanical thrombectomy with a stent retriever and/or thrombus aspiration, with or without local administration of a thrombolytic agent. The actual EVT strategy was at the discretion of the interventionist. Time of stroke onset was defined as the time of witnessed onset of symptoms or, if this was unknown, the moment that the patient was last known to be well.

Our primary clinical outcome measure was good functional outcome at 90 days post-stroke, defined as a score of 0–2 on the modified Rankin Scale (mRS). Other clinical outcome measures were the overall shift in mRS score between groups, occurrence of symptomatic intracranial hemorrhage (sICH) and mortality at 90 days post-stroke. Intracranial hemorrhage was defined as symptomatic if the patient died or deteriorated neurologically (an increase of ≥ 4 points on the National Institutes of Health Stroke Scale [NIHSS]) as a result of the hemorrhage [7]. Successful reperfusion, defined as a score of ≥ 2b on the extended thrombolysis in cerebral infarction (eTICI) scale, was used as a radiological outcome measure.

Workflow-related outcome measures were: time from stroke onset to arterial puncture [onset-to-groin time (OGT)], which was our main secondary outcome measure, and time from arrival at the first hospital to arterial puncture [first-door-to-groin time (FDGT)].

Transferred patients generally live farther away from a CSC than mothership patients, which makes a direct comparison of treatment times inherently biased in favor of patients directly presented to a CSC. To account for this bias, we calculated adjusted OGT and FDGT, in which we corrected for travel time by subtracting estimated ambulance travel times between the PSC and the CSC from the original treatment times, for all transferred patients. These data were provided by the Dutch National Institute for Public Health and the Environment and calculated using their proprietary model, assuming daytime circumstances outside of rush hour and the ambulance driving with the highest level of emergency [8].

### Statistical analysis

Patients who were directly presented to a CSC were compared to patients who were transferred from a PSC. We compared baseline characteristics using independent sample *t* test for normally distributed continuous variables, Mann–Whitney *U* test for non-normally distributed continuous variables, and *χ*^2^ test for categorical variables. For the regression analyses, we imputed missing data using multiple imputation, using the following covariates: age, sex, previous stroke, previous diabetes, previous atrial fibrillation, previous myocardial infarction, pre-stroke mRS score, baseline blood pressure (systolic and diastolic), baseline NIHSS score, location of occlusion, collateral status, onset-to-first-door time, OGT, FDGT, onset-to-reperfusion time, eTICI score after EVT, and mRS score at 90 days post-stroke. To analyze the odds of good functional outcome, defined as mRS 0–2 at 90 days, we used binary logistic regression. Ordinal logistic regression was used to assess the overall shift in mRS score between groups. In both analyses, we adjusted for the following pre-specified prognostic variables: age, pre-stroke mRS, anticoagulation use, baseline NIHSS, location of occlusion, collateral status, and onset-to-first-door time. For our analyses of sICH and mortality, we also used binary logistic regression, adjusting for age, pre-stroke mRS, anticoagulation use, baseline systolic blood pressure, baseline NIHSS, location of occlusion, collateral status and onset-to-first-door time. For analyzing successful reperfusion rate (eTICI ≥ 2b), we used binary logistic regression and adjusted for the following variables: age, location of occlusion and onset-to-first door time. Linear regression was used for the analyses of OGT and FDGT (with and without correction for travel time). In the OGT analysis, we adjusted for age, pre-stroke mRS, baseline blood pressure, baseline NIHSS, location of occlusion and presentation outside the 4.5 h time window. In the FDGT analysis, we adjusted for age, pre-stroke mRS, baseline blood pressure, baseline NIHSS, location of occlusion, and onset-to-first-door time. To explore residual confounding, we performed a secondary analysis in which we stratified for presentation to the first hospital within the 4.5 h time window. In this analysis, we used all clinical, radiological, and workflow-related outcomes named above. All analyses were performed using SPSS (version 25; SPSS Inc., Chicago, IL, USA).

## Results

**Fig. 1:**
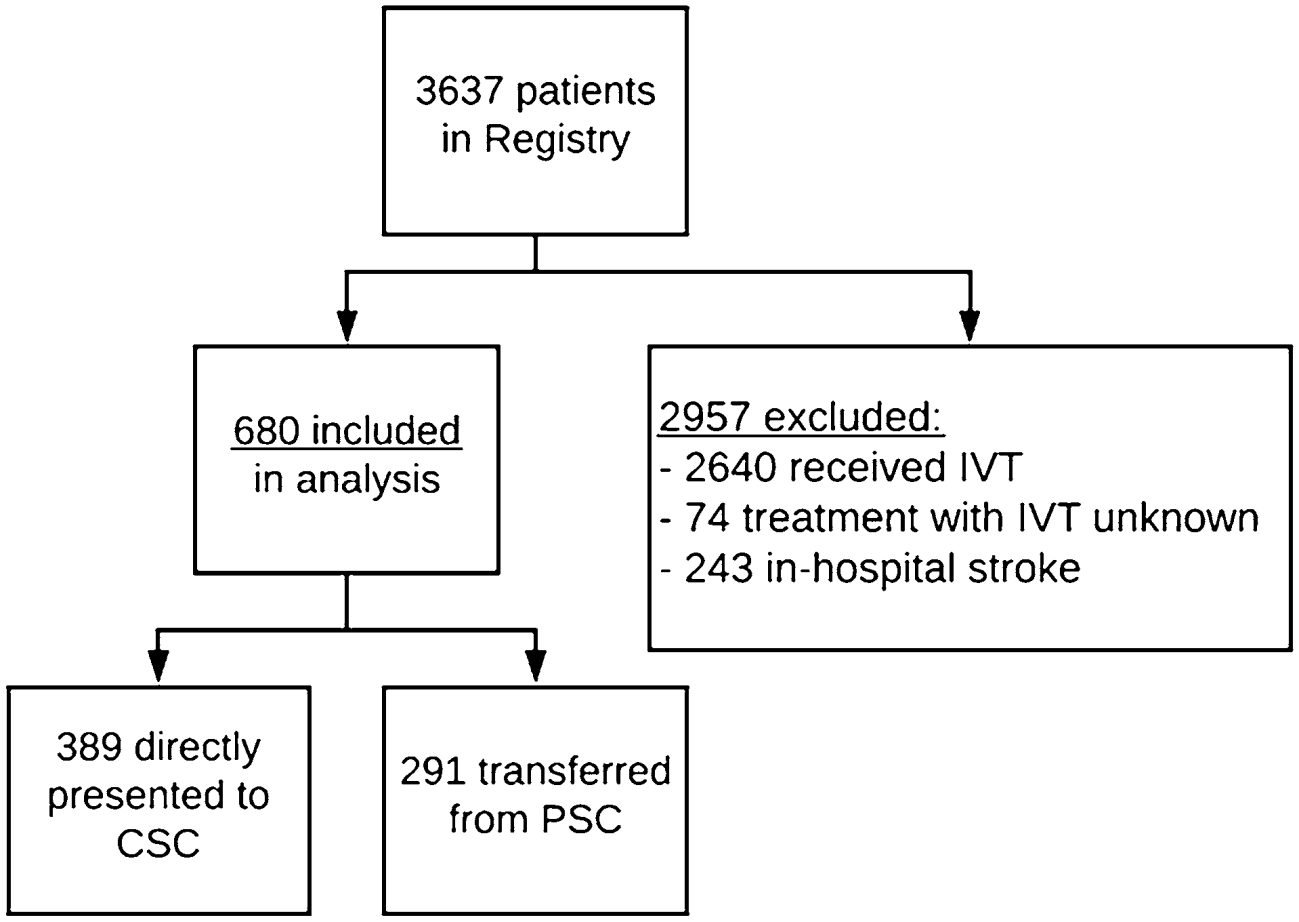
Flowchart of patient selection. *CSC* comprehensive stroke center, *IVT* intravenous thrombolysis, *Registry* the multicenter collaboration for endovascular treatment of acute ischemic stroke in the Netherlands (MR CLEAN Registry)

Between March 2014 and November 2017, 3637 patients were included in the MR CLEAN Registry (part I and II). We excluded 2957 patients, either because they were treated with IVT (*n* = 2640), because it was unknown whether they were treated with IVT (*n* = 74), or because they had an in-hospital stroke (*n* = 243). Therefore, 680 patients were included in the analysis (Fig. 1). Of these, 389 (57%) were directly presented to a CSC and 291 (43%) were transferred from a PSC. Patients who were directly presented to a CSC less often had atrial fibrillation (38% vs. 54%, *p* < 0.001), had lower baseline NIHSS scores (median 16 vs. 17, *p* = 0.015) and had higher collateral scores on baseline CTA (*p* = 0.003) compared to transferred patients. Onset-to-first-door times were longer for the direct group [median 105 vs. 66 min, *p* < 0.001 (Table 1)]. The median estimated ambulance travel time between PSC and CSC for the transferred group was 17 min (IQR: 10–31). Contraindications for IVT are listed in Table 1. Presentation beyond the 4.5 h time window was more common in directly presented patients (35% vs. 15%, *p* < 0.001). Use of a vitamin K antagonist was less frequent in directly presented patients (32% vs. 45%, *p* < 0.001), while heparin use (therapeutic dosage) was more common in the direct group (2% vs. 0%, *p* = 0.040). Other contraindications did not differ between the two groups.

**Table 1:** Baseline characteristics

	Direct, *n* = 389	Transfer, *n* = 291	*p* value
Age, years mean ± SD	71 ± 14.2	73 ± 12.5	0.053
Male sex, no./total (%)	181/389 (47%)	148/291 (51%)	0.264
Hypertension, no./total (%)	206/380 (54%)	172/287 (60%)	0.140
Diabetes mellitus, no./total (%)	59/386 (15%)	41/289 (14%)	0.691
Atrial fibrillation, no./total (%)	147/384 (38%)	154/286 (54%)	** < 0.001**
Myocardial infarction, no./total (%)	55/385 (14%)	54/278 (19%)	0.078
Previous stroke, no./total (%)	103/387 (27%)	80/287 (28%)	0.667
Pre-stroke mRS score^a^, median (IQR)	0 (0–2)	0 (0–1)	0.180
Systolic blood pressure^b^, mean ± SD	154 ± 28.0	152 ± 26.9	0.395
Diastolic blood pressure^c^, mean ± SD	84 ± 17.0	83 ± 16.7	0.897
NIHSS scored, median (IQR)	16 (10–20)	17 (13–21)	**0.015**
Occlusion site, no./total (%)			0.119
ICA	58/342 (17%)	59/268 (22%)	
M1	169/342 (49%)	138/268 (52%)	
M2	58/342 (17%)	44/268 (16%)	
Anterior cerebral artery	4/342 (1%)	1/268 (0%)	
Posterior circulation	34/342 (10%)	14/268 (5%)	
ASPECTS score on first NCCT^e^, median (IQR)	9 (7–10)	9 (7–10)	0.513
Collateral score on first CTA, no./total (%)			**0.003**
Grade 0	20/311 (6%)	19/256 (7%)	
Grade 1	100/311 (32%)	105/256 (41%)	
Grade 2	108/311 (35%)	96/256 (38%)	
Grade 3	83/311 (27%)	36/256 (14%)	
Time from stroke onset to door of first hospital, minutes^f^, median (IQR)	105 (51–266)	66 (40–132)	** < 0.001**
Contraindication for IVT, no./total (%)			
Use of vitamin K antagonist	108/336 (32%)	118/262 (45%)	** < 0.001**
Presentation > 4.5 h	118/336 (35%)	39/262 (15%)	** < 0.001**
Recent clinical event^g^	56/336 (17%)	51/262 (19%)	0.267
Use of DOAC	30/336 (9%)	34/262 (13%)	0.079
Hypertension	10/336 (3%)	10/262 (4%)	0.509
Unfavorable characteristics NCCT	8/336 (2%)	6/262 (2%)	0.996
Use of heparin in therapeutic dosage	6/336 (2%)	0/262 (0%)	**0.040**^**h**^
Other	9/336 (3%)	11/262 (4%)	0.263

Statistically significant findings are displayed in bold

*ASPECTS* Alberta Stroke Program Early CT Score; *CTA* computed tomography angiography; *DOAC* direct oral anticoagulant; *ICA* intracranial part of internal carotid artery; *IQR* interquartile range; *IVT* intravenous thrombolysis; *M1* first segment of the middle cerebral artery; *M2* second segment (after first bifurcation) of the middle cerebral artery; *mRS* modified Rankin Scale; *NCCT* non-contrast computed tomography; *NIHSS* National Institutes of Health Stroke Scale; *no.* number; *SD* standard deviation

Number of missing values: ^a^14; ^b^19; ^c^27; ^d^12; ^e^91; ^f^96

^g^Recent hemorrhagic or ischemic stroke, recent major surgery, recent gastrointestinal or urogenital bleeding or recent head trauma

^h^Fisher’s exact test was used for this analysis

**Fig. 2:**
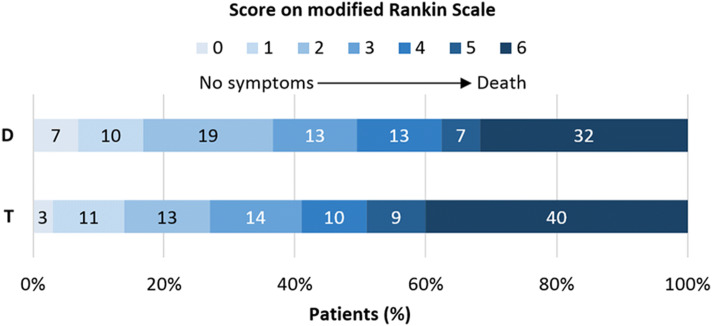
Functional outcome according to modified Rankin Scale score at 90 days post-stroke. *D* direct group, *T* transferred group

Functional outcome was slightly better in patients who were directly presented to a CSC [mRS 0–2: 36 vs. 28%, OR: 1.51, 95% CI 1.06–2.15 (Table 2; Fig. 2)]. After adjustment, statistical significance was lost (adjusted OR: 1.23, 95% CI 0.73–2.08). When analyzing the shift in overall mRS scores between groups, the results were similar (unadjusted common OR: 1.43, 95% CI 1.07–1.91; adjusted common OR: 1.21, 95% CI 0.80–1.84). Incidence of sICH did not differ between the direct group and the transferred group (5% vs. 5%; adjusted OR: 0.70, 95% CI 0.28–1.75). Other clinical and radiological outcomes also were not different (Table 2).

**Table 2:** Clinical and radiological outcomes

	Direct, *n* = 389	Transfer, *n* = 291	Unadjusted OR^a^ (95% CI)	Adjusted OR^a^ (95% CI)
Functional independence at 90 days (mRS 0–2), no./total (%)	130/360 (36%)	73/263 (28%)	**1.51 (1.06–2.15)**	1.23 (0.73–2.08)^b^
mRS score at 90 days^c^, median (IQR)	4 (2–6)	4 (2–6)	**1.43 (1.07–1.91)**^d^	1.21 (0.80–1.84)^b^
Symptomatic intracranial hemorrhage, no./total (%)	20/388 (5%)	16/291 (5%)	0.93 (0.47–1.83)	0.70 (0.28–1.75)^e^
Mortality at 90 days, no./total (%)	116/360 (32%)	105/263 (40%)	**0.71 (0.51–0.98)**	0.90 (0.54–1.50)^e^
Successful reperfusion (eTICI ≥ 2b), no./total (%)	186/321 (60%)	156/271 (58%)	0.89 (0.61–1.30)	1.15 (0.78–1.68)^f^

Statistically significant findings are displayed in bold

*CI* confidence interval; *eTICI* extended thrombolysis in cerebral infarction scale; *IQR* interquartile range, *mRS* modified Rankin Scale; *no.* number; *OR* odds ratio

^a^Odds for the direct group

^b^Adjusted for age, pre-stroke mRS, anticoagulation use, baseline NIHSS, location of occlusion, collateral status and onset-to-first-door time

^d^Odds of 1-point shift towards a favorable outcome on the mRS for the direct group

^e^Adjusted for age, pre-stroke mRS, anticoagulation use, baseline systolic blood pressure, baseline NIHSS, location of occlusion, collateral status, and onset-to-first-door time

^f^Adjusted for age, location of occlusion, and onset-to-first door time

Number of missing values: ^c^57

Patients directly presented to a CSC had a median OGT of 220 min, compared to 230 min for transferred patients [adjusted *β* = − 40.0, 95% CI − 61.5 to − 18.5 (Table 3)]. When the increased travel time for transferred patients was taken into account, adjusted OGT was still 18 min shorter for patients directly presented to a CSC, although this was not statistically significant (median 220 vs. 207 min; adjusted *β* = − 18.1, 95% CI − 39.6 to 3.4). FDGT was also shorter for the direct group (median 93 vs. 150 min; adjusted *β* = − 51.0, 95% CI − 64.1 to − 37.9), even when corrected for increased travel time (median 93 vs. 127 min, adjusted *β* = − 28.0, 95% CI − 41.3 to − 14.7).

**Table 3:** Treatment times

	Direct, *n* = 389	Transfer, *n* = 291	Unadjusted *β* (95% CI)	Adjusted *β* (95% CI)
Onset-to-groin time^a^, median (IQR)	220 (143–360)	230 (283–320)	16.5 (− 9.0 to 42.0)	**− 40.0 (− 61.5 to -18.5)**^b^
Travel time-corrected onset-to-groin time^c^, median (IQR)	220 (143–360)	207 (163–293)	**37.4 (12.0 to 62.8)**	− 18.1 (− 39.6 to 3.4)^b^
First-door-to-groin time^d^, median (IQR)	93 (72–125)	150 (115–186)	**− 41.7 (− 52.1 to − 31.4)**	**− 51.0 (− 64.1 to − 37.9)**^e^
Travel time-corrected first-door-to-groin time^f^, median (IQR)	93 (72–125)	127 (96–166)	**− 20.9 (− 31.2 to − 10.6)**	**− 28.0 (− 41.3 to − 14.7)**^e^

Statistically significant findings are displayed in bold

*CI* confidence interval; *IQR* interquartile range

^**b**^Adjusted for age, pre-stroke mRS, baseline blood pressure, baseline NIHSS, location of occlusion, and presentation outside the 4.5 h time window

^e^Adjusted for age, pre-stroke mRS, baseline blood pressure, baseline NIHSS, location of occlusion, and onset-to-first-door time

Number of missing values: ^a^11; ^c^24; ^d^95; ^f^100

We stratified the analysis for presentation to the first hospital within the 4.5 h time window. Of 476 patients presented within 4.5 h, 290 (61%) were presented directly to a CSC. Among patients presented outside the 4.5 h time window, direct presentation was more common, with 90/108 patients (83%) being presented directly to a CSC. Baseline characteristics of the two strata are shown in Online Resource 2. Within the 4,5 h time window, patients presented directly to a CSC more often were functionally independent at 90 days [mRS 0–2: 36 vs. 26%, OR: 1.65, 95% CI 1.07–2.56 (Table 4)]. After adjustments for potential confounders, this difference was no longer statistically significant (adjusted OR: 1.28, 95% CI 0.74–2.22). We found similar results for the overall shift in mRS between groups among patients presented within 4.5 h (unadjusted common OR: 1.47, 95% CI 1.04–2.09; adjusted common OR: 1.17, 95% CI 0.76–1.81). Among patients who were presented > 4.5 h after symptom onset there was no difference in functional outcome (adjusted OR: 0.89, 95% CI 0.18–4.29). Other clinical and radiological outcomes also did not differ (Table 4). In patients presented within 4.5 h, the difference in OGT remained statistically significant, in favor of the direct group (median 180 vs. 228 min; adjusted *β* = − 46.4, 95% CI − 66.1 to − 26.6). In patients presented after > 4.5 h, OGT did not differ between groups (median 457 vs. 455 min; adjusted *β* = − 8.1, 95% CI − 115.9 to 99.7). The difference in FDGT in favor of the direct group remained statistically significant in both strata (≤ 4.5 h: adjusted *β* = − 50.8, 95% CI − 65.7 to − 36.2; > 4.5 h: adjusted *β* = − 47.0, 95% CI − 71.7 to − 22.3). The results of our analyses for both strata of OGT and FDGT corrected for travel time are reported in Online Resource 3.

**Table 4:** Clinical and radiological outcomes stratified by presentation within 4.5 h time window

	Presentation ≤ 4.5 h				Presentation > 4.5 h			
	Direct, *n* = 290	Transfer, *n* = 186	Unadjusted OR^a^ (95% CI)	Adjusted OR^a^ (95% CI)	Direct, *n* = 90	Transfer, *n* = 18	Unadjusted OR^a^ (95% CI)	Adjusted OR^a^ (95% CI)
Functional independence at 90 days (mRS 0–2) no./total (%)	95/265 (36%)	46/175 (26%)	**1.65 (1.07–2.56)**	1.28 (0.74–2.22)^b^	33/87 (38%)	6/16 (38%)	1.12 (0.38–3.36)	0.89 (0.18–4.29)^b^
mRS score at 90 days^c^ median (IQR)	4 (2–6)	4 (2–6)	**1.47 (1.04–2.09)**^d^	1.17 (0.76–1.81)^b^	4 (2–6)	4 (2–6)	1.20 (0.47–3.11)^d^	1.15 (0.32–4.13)^b^
sICH – no./total (%)	14/290 (5%)	11/186 (6%)	0.81 (0.36–1.82)	0.66 (0.25–1.72)^e^	4/90 (4%)	0/18 (0%)	–	–
Mortality at 90 days no. (%)	90/265 (34%)	68/175 (39%)	0.76 (0.51–1.12)	0.94 (0.55–1.61)^e^	23/87 (26%)	5/16 (31%)	0.80 (0.25–2.59)	0.56 (0.09–3.50)^e^
Successful reperfusion (eTICI ≥ 2b) no. (%)	145/240 (60%)	96/174 (55%)	1.07 (0.69–1.68)	1.19 (0.78–1.79)^f^	37/77 (48%)	5/15 (33%)	1.65 (0.54–5.03)	1.26 (0.38–4.18)^f^

Statistically significant findings are displayed in bold

*CI* confidence interval; *eTICI* extended thrombolysis in cerebral infarction scale; *IQR* interquartile range, *mRS* modified Rankin Scale; *no.* number; *OR* odds ratio; *sICH* symptomatic intracranial hemorrhage

^a^Odds for the direct group

^b^Adjusted for age, pre-stroke mRS, anticoagulant use, baseline NIHSS, location of occlusion, and collateral status

^d^Odds of 1-point shift towards a favorable outcome on the mRS at 90 days for the direct group

^e^Adjusted for age, pre-stroke mRS, anticoagulant use, baseline systolic blood pressure, baseline NIHSS, location of occlusion, and collateral status

^f^Adjusted for age and location of occlusion

Number of missing values: ^c^41

## Discussion

In this nationwide cohort of patients who underwent EVT for AIS, we found that in the subgroup of patients who were not eligible for IVT, treatment times were shorter for patients directly presented to a CSC, compared to patients who were first presented to a PSC. Clinical outcome was also slightly better in directly presented patients, although this was not statistically significant.

Previous post hoc analyses of prospective cohort studies have shown that for patients with an LVO of the anterior circulation, in general, it is beneficial to be directly presented to a CSC, as opposed to being transferred from a PSC. Venema et al., who also used data from the MR CLEAN Registry (part I), found that patients directly presented to a CSC had a 40-min shorter OGT and a 57-min shorter FDGT than transferred patients [3]. The authors also found a negative effect of inter-hospital transfer on the likelihood of functioning independently at 90 days post-stroke (OR: 0.69, 95% CI 0.54–0.89). A post hoc analysis of data from the STRATIS Registry (Systematic Evaluation of Patients Treated With Neurothrombectomy Devices for Acute Ischemic Stroke) showed a similar beneficial effect of direct presentation on functional outcome [5]. However, in a subgroup analysis of patients who were not treated with IVT, the authors found that despite the OGT being almost an hour lower, the chance of good functional outcome did not differ between directly presented and transferred patients (56% vs. 50%, *p* = 0.23). Since previous studies have convincingly shown that earlier initiation of EVT improves clinical outcome, these findings seem discrepant [4, 9–11]. However, the authors did not report if and how their subgroup analysis was adjusted for possible confounders, neither were baseline characteristics reported for the subgroup of patients treated with EVT alone.

In the current study, despite adjustment for baseline imbalances, we found the same discrepancy as did the authors of the STRATIS Registry substudy: a beneficial effect of direct presentation on time to treatment, but no statistically significant difference in functional outcome. A potential explanation for this finding is that our sample size was too small to find a difference in functional outcome, but substantial enough to show the larger differences in time to treatment. Another possible explanation could be residual confounding. Although we tried to adjust for factors that, based on baseline characteristics and clinical experience, may have influenced the hospital choice by ambulance paramedics or the choice of referral for EVT by PSC neurologists, there may be other confounding factors that we did not take into account or that we had no data for. For example, patients with severe comorbidity (e.g. active malignancy, renal failure, and congestive heart failure) may be more likely to be directly presented to CSCs, since CSCs are often tertiary care centers. Because data of these severe comorbidities were not available for our study, we could not adjust our analyses for this potential confounder.

There were some baseline imbalances between the direct and the transferred group that warrant mention. First, the transferred group more often had atrial fibrillation. A probable explanation is that this is due to the different distributions of contraindications for IVT over the two groups. Since the transfer group contains relatively few patients in the > 4.5 h time window, other contraindications for IVT are more prevalent in this group. Of these, the most common contraindication is use of anticoagulant medication. Because the indication for anticoagulation use often is atrial fibrillation, this may explain the higher prevalence in this group. Second, the transferred group had higher baseline NIHSS scores. A possible explanation could be that patients with relatively mild neurological deficits were less often referred from a PSC, for instance because the deficits were not considered to be sufficiently severe to warrant EVT. Third, collateral scores were slightly better in directly presented patients. This may be because atrial fibrillation was less common in this group, since this has been associated with worse collaterals [12]. Fourth, time from stroke onset to arrival at the first hospital was significantly longer for directly presented patients. Most likely, this is because ambulance paramedics were inclined to bring patients who were (almost) outside the 4.5 h time window, who were thus only eligible for EVT, directly to a CSC.

We specifically focused on the effects of interhospital transfer in the subgroup of patients not eligible for IVT. We chose to do so, because of the relevance of this subject for routine clinical practice. In approximately 15–20% of patients with AIS, a contraindication for IVT is present [2, 6]. Unlike patients eligible for IVT, in whom direct presentation to a CSC may delay initiation of this treatment, patients ineligible for IVT have no major disadvantage of being presented directly to a CSC. Moreover, the most common contraindications that render patients ineligible for IVT could be identified by ambulance paramedics. For example, anticoagulant use or duration of symptoms could be determined through a patient history. Blood pressure is routinely measured, and an INR could be determined using a point of care test [13]. Further study on this issue is required, for instance to ascertain if determining IVT contraindications negatively influences ambulance response times. However, if such studies do not show any major negative effects, prehospital triage of this patient group could relatively easily be implemented.

Some limitations of our study should also be considered. First of all, the Netherlands, where data collection for this study took place, is a relatively small and densely populated country, where hospitals are located relatively close to one another [14]. Therefore, the differences in time to treatment between directly presented and transferred patients that we found in this study, are likely smaller than they would have been in less densely populated areas [15, 16]. Consequently, our findings should be extrapolated to other countries with caution.

Second, it is likely that our data were affected by selection bias. In the MR CLEAN Registry, patients with an LVO who were ultimately deemed ineligible for EVT for whatever reason, were not included. As a consequence, we have no data of patients that could not receive EVT because of time lost by primary transportation to a PSC causing the time window for EVT to exceed [17]. Therefore, the negative effects of inter-hospital transfer may be larger than shown in this study. Additionally, selection bias is inherent to the manner of hospital selection by ambulance paramedics. Even though the protocol in the Netherlands is to bring patients with a suspected stroke to the nearest stroke center, ambulance paramedics may nonetheless decide to bypass a PSC and bring a patient directly to a CSC. In a similar way, factors affecting the decision of PSC neurologists whether or not to refer a patient for EVT may have influenced our data. By adjusting our analyses for potential confounders, based on clinical experience and baseline imbalances, we have tried to minimize the impact of this issue on our results.

Finally, for some variables we had relatively high numbers of missing values, the most important of which were onset-to-first-door time (14%), FDGT (14%), and mRS score at 90 days post-stroke (8%). We tried to minimize the impact of the missing data on our analyses using multiple imputation, as described in the methods section.

Further research should focus on finding a triage instrument for prehospital selection of patients eligible for EVT, so that these patients can be brought directly to a CSC, without overburdening these hospitals with patients who can be treated in a PSC. Results of other research toward optimization of prehospital stroke logistics are expected in the coming years: a randomized controlled trial in Spain [Direct Transfer to an Endovascular Center Compared to Transfer to the Closest Stroke Centre in Acute Stroke Patients With Suspected Large Vessel Occlusion (RACECAT), Clinicaltrials.gov number: NCT02795962] is comparing direct presentation to transfer to a CSC in patients with a high likelihood of an LVO. Until then, directly presenting patients with a suspected stroke and a contraindication for IVT to a CSC may be considered, since there is no obvious disadvantage in bypassing the PSC in this patient population. Implementing this would, however, result in a higher patient load for CSCs. Other studies have shown that approximately 10% of suspected stroke patients have an LVO [18], and in our cohort, 19% of patients with an LVO had a contraindication for IVT. Assuming that the proportion of patients with a contraindication for IVT is also 19% in the entire population of patients with a suspected AIS, routing these patients directly to a CSC would mean that for every patient with an LVO and a contraindication for IVT, approximately nine patients without an LVO and with a contraindication for IVT would be presented to a CSC. In addition to the higher patient load, unnecessary transportation of these patients to a CSC may also be a burden on the patients and their families, because it may involve admission to a hospital further from home. To reduce the number of unnecessary direct presentations to a CSC, a triage method applied by paramedics, such as a clinical LVO-detection scale, may be useful.

In conclusion, we showed that in patients with an LVO who were not eligible for IVT, direct presentation to a CSC decreased time to EVT, compared to initial presentation to a PSC. Direct presentation was also associated with a slightly better clinical outcome that was not statistically significant. Since there is no obvious disadvantage in bypassing the PSC in this patient population, directly presenting patients with a suspected stroke and a contraindication for IVT to a CSC, if logistically feasible, may be considered.

## Electronic supplementary material

Below is the link to the electronic supplementary material.

Supplementary file1 (PDF 98 kb)

Supplementary file2 (PDF 177 kb)

Supplementary file3 (PDF 158 kb)
