# Supplementary material for: Interhospital transfer vs. direct presentation of patients with a large vessel occlusion not eligible for IV thrombolysis
Source: J Neurol. 2020 Apr 7;267(7):2142–50. doi: 10.1007/s00415-020-09812-5 (PMC7320925; doi:10.1007/s00415-020-09812-5)
Supplement: Supplementary file 1 — Supplementary file1 (PDF 98 kb) [file 415_2020_9812_MOESM1_ESM.pdf]

ELECTRONIC SUPPLEMENTAL MATERIAL: ONLINE RESOURCE 1

TRANSFER VS. DIRECT PRESENTATION TO A COMPREHENSIVE STROKE CENTER OF PATIENTS WITH A LARGE VESSEL OCCLUSION NOT ELIGIBLE FOR IV THROMBOLYSIS

Laura C.C. van Meenen[1], Adrien E. Groot[1], Esmee Venema[2], Bart J. Emmer[3], Martin D. Smeekes[4], Geert Jan Kommer[5], Charles B.L.M. Majoie[3], Yvo B.W.E.M. Roos[1], Wouter J. Schonewille[6], Bob Roozenbeek[7], Jonathan M. Coutinho[1] on behalf of the MR CLEAN Registry Investigators[8]

1. Department of Neurology, Amsterdam University Medical Centers, University of Amsterdam, the Netherlands
2. Department of Neurology and Department of Public Health, Erasmus MC University Medical Center, Rotterdam, the Netherlands
3. Department of Radiology and Nuclear Medicine, Amsterdam University Medical Centers, University of Amsterdam, the Netherlands
4. Emergency Medical Services North-Holland North, Alkmaar, the Netherlands
5. National Institute of Public Health and the Environment, Center for Nutrition, Prevention and Health services
6. Department of Neurology, St. Antonius Ziekenhuis, Nieuwegein, the Netherlands
7. Department of Radiology & Nuclear Medicine, Erasmus MC University Medical Center, Rotterdam, the Netherlands
8. MR CLEAN Registry Investigators - group authors: please see Online Resource 1

Corresponding author: dr. J.M. Coutinho (telephone: +31 20 732 2289, email: j.coutinho@amsterdamumc.nl)

Online Resource 1. MR CLEAN Registry Investigators - group authors

Executive committee

Diederik W.J. Dippel[1];Aad van der Lugt[2];Charles B.L.M. Majoie[3];Yvo B.W.E.M. Roos[4];Robert J. van Oostenbrugge[5];Wim H. van Zwam[6];Jelis Boiten[14];Jan Albert Vos[8]

Study coordinators

Josje Brouwer [4];Sanne J. den Hartog[1,2,40];Wouter H. Hinsenveld [5,6];Manon Kappelhof[3];Kars C.J. Compagne[2];Robert- Jan B. Goldhoorn[5,6]; Maxim J.H.L. Mulder[1,2]; Ivo G.H. Jansen[3]

Local principal investigators

Diederik W.J. Dippel[1];Bob Roozenbeek[1];Aad van der Lugt[2];Adriaan C.G.M. van Es[2];Charles B.L.M. Majoie[3];Yvo B.W.E.M. Roos[4];Bart J. Emmer[3];Jonathan M. Coutinho[4];Wouter J. Schonewille[7];Jan Albert Vos[8]; Marieke J.H. Wermer[9];Marianne A.A. van Walderveen[10];Julie Staals[5];Robert J. van Oostenbrugge[5];Wim H. van Zwam[6];Jeannette Hofmeijer[11];Jasper M. Martens[12];Geert J. Lycklama à Nijeholt[13];Jelis Boiten[14];Sebastiaan F. de Bruijn[15];Lukas C. van Dijk[16];H. Bart van der Worp[17];Rob H. Lo[18];Ewoud J. van Dijk[19];Hieronymus D. Boogaarts[20];J. de Vries[22];Paul L.M. de Kort[21]; Julia van Tuijl[21] ;Jo Jo P. Peluso[26];Puck Fransen[22];Jan S.P. van den Berg[22];Boudewijn A.A.M. van Hasselt[23];Leo A.M. Aerden[24];René J. Dallinga[25];Maarten Uyttenboogaart[28];Omid Eschgi[29];Reinoud P.H. Bokkers[29];Tobien H.C.M.L. Schreuder[30];Roel J.J. Heijboer[31];Koos Keizer[32];Lonneke S.F. Yo[33];Heleen M. den Hertog[22];Emiel J.C. Sturm[35]; Paul Brouwers[34]

Imaging assessment committee

Charles B.L.M. Majoie[3](chair);Wim H. van Zwam[6];Aad van der Lugt[2];Geert J. Lycklama à Nijeholt[13];Marianne A.A. van Walderveen[10];Marieke E.S. Sprengers[3];Sjoerd F.M. Jenniskens[27];René van den Berg[3];Albert J. Yoo[38];Ludo F.M. Beenen[3];Alida A. Postma[6];Stefan D. Roosendaal[3];Bas F.W. van der Kallen[13];Ido R. van den Wijngaard[13];Adriaan C.G.M. van Es[2];Bart J. Emmer[,3];Jasper M. Martens[12]; Lonneke S.F. Yo[33];Jan Albert Vos[8]; Joost Bot[36], Pieter-Jan van Doormaal[2]; Anton Meijer[27];Elyas Ghariq[13]; Reinoud P.H. Bokkers[29];Marc P. van Proosdij[37];G. Menno Krietemeijer[33];Jo P. Peluso[26];Hieronymus D. Boogaarts[20];Rob Lo[18];Dick Gerrits[35];Wouter Dinkelaar[2]Auke P.A. Appelman[29];Bas Hammer[16];Sjoert Pegge[27];Anouk van der Hoorn[29];Saman Vinke[20].

Writing committee

Diederik W.J. Dippel[1](chair);Aad van der Lugt[2];Charles B.L.M. Majoie[3];Yvo B.W.E.M. Roos[4];Robert J. van Oostenbrugge[5];Wim H. van Zwam[6];Geert J. Lycklama à Nijeholt[13];Jelis Boiten[14];Jan Albert Vos[8];Wouter J. Schonewille[7];Jeannette Hofmeijer[11];Jasper M. Martens[12];H. Bart van der Worp[17];Rob H. Lo[18]

Adverse event committee

Robert J. van Oostenbrugge[5](chair);Jeannette Hofmeijer[11];H. Zwenneke Flach[23]

Trial methodologist

Hester F. Lingsma[40]

Research nurses / local trial coordinators

Naziha el Ghannouti[1];Martin Sterrenberg[1];Corina Puppels[7];Wilma Pellikaan[7];Rita Sprengers[4];Marjan Elfrink[11];Michelle Simons[11];Marjolein Vossers[12];Joke de Meris[14];Tamara Vermeulen[14];Annet Geerlings[19];Gina van Vemde[22];Tiny Simons[30];Cathelijn van Rijswijk[21];Gert Messchendorp[28];Nynke Nicolaij[28];Hester Bongenaar[32];Karin Bodde[24];Sandra Kleijn[34];Jasmijn Lodico[34]; Hanneke Droste[34];Maureen Wollaert[5];Sabrina Verheesen[5];D. Jeurrissen[5];Erna Bos[9];Yvonne Drabbe[15];Michelle Sandiman[15];Marjan Elfrink[11];Nicoline Aaldering[11];Berber Zweedijk[17];Mostafa Khalilzada[15];Jocova Vervoort[21];Hanneke Droste[34];Nynke Nicolaij[2];Michelle Simons[11];Eva Ponjee[22];Sharon Romviel[19];Karin Kanselaar[19];Erna Bos[9];Denn Barning[10].

PhD / Medical students

Esmee Venema[40]; Vicky Chalos[1,40];Ralph R. Geuskens[3]; Tim van Straaten[19];Saliha Ergezen[1]; Roger R.M. Harmsma[1]; Daan Muijres[1]; Anouk de Jong[1];Olvert A. Berkhemer[1,3,6];Anna M.M. Boers[3,39]; J. Huguet[3];P.F.C. Groot[3];Marieke A. Mens[3];Katinka R. van Kranendonk[3];Kilian M. Treurniet[3];Ivo G.H. Jansen[3];Manon L. Tolhuisen[3,39];Heitor Alves[3];Annick J. Weterings[3],Eleonora L.F. Kirkels[3],Eva J.H.F. Voogd[11];Lieve M. Schupp[3];Sabine Collette[28,29];Adrien E.D. Groot[4];Natalie E. LeCouffe[4];Praneeta R. Konduri[39];Haryadi Prasetya[39];Nerea Arrarte-Terreros[39];Lucas A. Ramos[39].

List of affiliations

Department of Neurology[1], Radiology[2], Public Health[40], Erasmus MC University Medical Center;

Department of Radiology and Nuclear Medicine[3], Neurology[4], Biomedical Engineering & Physics[39], Amsterdam UMC, University of Amsterdam, Amsterdam;

Department of Neurology[5], Radiology[6], Maastricht University Medical Center and Cardiovascular Research Institute Maastricht (CARIM);

Department of Neurology[7], Radiology[8], Sint Antonius Hospital, Nieuwegein;

Department of Neurology[9], Radiology[10], Leiden University Medical Center;

Department of Neurology[11], Radiology[12], Rijnstate Hospital, Arnhem;

Department of Radiology[13], Neurology[14], Haaglanden MC, the Hague;

Department of Neurology[15], Radiology[16], HAGA Hospital, the Hague;

Department of Neurology[17], Radiology[18], University Medical Center Utrecht;

Department of Neurology[19], Neurosurgery[20], Radiology[27], Radboud University Medical Center, Nijmegen;

Department of Neurology[21], Radiology[26], Elisabeth-TweeSteden ziekenhuis, Tilburg;

Department of Neurology[22], Radiology[23], Isala Klinieken, Zwolle;

Department of Neurology[24], Radiology[25], Reinier de Graaf Gasthuis, Delft;

Department of Neurology[28], Radiology[29], University Medical Center Groningen;

Department of Neurology[30], Radiology[31], Atrium Medical Center, Heerlen;

Department of Neurology[32], Radiology[33], Catharina Hospital, Eindhoven;

Department of Neurology[34], Radiology[35], Medical Spectrum Twente, Enschede;

Department of Radiology[36], Amsterdam UMC, Vrije Universiteit van Amsterdam, Amsterdam; Department of Radiology[37], Noordwest Ziekenhuisgroep, Alkmaar;

Department of Radiology[38], Texas Stroke Institute, Texas, United States of America.
