# Supplementary material for: Interhospital transfer vs. direct presentation of patients with a large vessel occlusion not eligible for IV thrombolysis
Source: J Neurol. 2020 Apr 7;267(7):2142–50. doi: 10.1007/s00415-020-09812-5 (PMC7320925; doi:10.1007/s00415-020-09812-5)
Supplement: Supplementary file 2 — Supplementary file2 (PDF 177 kb) [file 415_2020_9812_MOESM2_ESM.pdf]

ELECTRONIC SUPPLEMENTAL MATERIAL: ONLINE RESOURCE 2

**TRANSFER VS. DIRECT PRESENTATION TO A COMPREHENSIVE STROKE CENTER OF PATIENTS WITH A LARGE VESSEL OCCLUSION NOT ELIGIBLE FOR IV THROMBOLYSIS**

Laura C.C. van Meenen[1], Adrien E. Groot[1], Esmee Venema[2], Bart J. Emmer[3], Martin D. Smeekes[4], Geert Jan Kommer[5], Charles B.L.M. Majoie[3], Yvo B.W.E.M. Roos[1], Wouter J. Schonewille[6], Bob Roozenbeek[7], Jonathan M. Coutinho[1] on behalf of the MR CLEAN Registry Investigators[8]

1. Department of Neurology, Amsterdam University Medical Centers, University of Amsterdam, the Netherlands
2. Department of Neurology and Department of Public Health, Erasmus MC University Medical Center, Rotterdam, the Netherlands
3. Department of Radiology and Nuclear Medicine, Amsterdam University Medical Centers, University of Amsterdam, the Netherlands
4. Emergency Medical Services North-Holland North, Alkmaar, the Netherlands
5. National Institute of Public Health and the Environment, Center for Nutrition, Prevention and Health services
6. Department of Neurology, St. Antonius Ziekenhuis, Nieuwegein, the Netherlands
7. Department of Radiology & Nuclear Medicine, Erasmus MC University Medical Center, Rotterdam, the Netherlands
8. MR CLEAN Registry Investigators - group authors: please see Online Resource 1

Corresponding author: dr. J.M. Coutinho (telephone: +31 20 732 2289, email: j.coutinho@amsterdamumc.nl)

**Online Resource 2. Baseline characteristics stratified by time of presentation within 4.5 hour time window**

| | Presentation ≤ 4.5 hours | | | Presentation > 4.5 hours | | |
|---|---|---|---|---|---|---|
| | **Direct, n=290** | **Transfer, n=186** | ***p* value** | **Direct, n=90** | **Transfer, n=18** | ***p* value** |
| **Age, years – mean ± SD** | 73 ± 13.0 | 74 ± 12.0 | 0.322 | 66 ± 16.3 | 65 ± 17.0 | 0.748 |
| **Male sex – no./total (%)** | 142/290 (49%) | 97/186 (52%) | 0.498 | 36/90 (40%) | 8/18 (44%) | 0.726 |
| **Hypertension – no./total (%)** | 156/283 (55%) | 115/184 (63%) | 0.114 | 47/90 (52%) | 8/18 (44%) | 0.547 |
| **Diabetes mellitus – no./total (%)** | 40/288 (14%) | 26/184 (14%) | 0.941 | 18/89 (20%) | 1/18 (6%) | 0.137 |
| **Atrial fibrillation – no./total (%)** | 130/287 (45%) | 109/183 (60%) | **0.003** | 17/88 (19%) | 1/18 (6%) | 0.156 |
| **Myocardial infarction – no./total (%)** | 43/287 (15%) | 31/180 (17%) | 0.519 | 10/89 (11%) | 1/18 (6%) | 0.469 |
| **Previous stroke – no./total (%)** | 87/290 (30%) | 53/184 (29%) | 0.781 | 14/89 (16%) | 4/18 (22%) | 0.502 |
| **Pre-stroke mRS score[a] – median (IQR)** | 0 (0-2) | 0 (0-1) | 0.243 | 0 (0-1) | 0 (0-0) | 0.171 |
| **Systolic blood pressure[b] – mean ± SD** | 154 ± 28.7 | 152 ± 26.4 | 0.510 | 153 ± 25.9 | 149 ± 36.8 | 0.656 |
| **Diastolic blood pressure[c] – mean ± SD** | 84 ± 17.3 | 85 ± 16.5 | 0.401 | 82 ± 15.2 | 82 ± 21.3 | 0.971 |
| **NIHSS score[d] – median (IQR)** | 15 (10-20) | 17 (12-21) | 0.073 | 16 (12-20) | 17 (13-23) | 0.228 |
| **Occlusion site – no./total (%)** | | | 0.156 | | | 0.421 |
| ICA | 39/290 (13%) | 34/186 (18%) | | 17/89 (19%) | 2/18 (11%) | |
| M1 | 124/290 (43%) | 91/186 (49%) | | 44/89 (49%) | 6/18 (33%) | |
| M2 | 47/290 (16%) | 29/186 (16%) | | 10/89 (11%) | 5/18 (28%) | |
| Anterior cerebral artery | 4/290 (1%) | 1/186 (1%) | | 0/89 (0%) | 0/18 (0%) | |

| | | | | | | |
|---|---|---|---|---|---|---|
| Posterior circulation | 22/290 (8%) | 7/186 (4%) | | 8/89 (9%) | 2/18 (11%) | |
| **ASPECTS score on first NCCT[e] – median (IQR)** | 9 (7-10) | 9 (8-10) | 0.277 | 8 (6-10) | 8 (6-9) | 0.614 |
| **Collateral score on first CTA – no./total (%)** | | | 0.180 | | | 0.094 |
| Grade 0 | 18/231 (8%) | 10/160 (6%) | | 2/77 (3%) | 0/15 (0%) | |
| Grade 1 | 78/231 (34%) | 64/160 (40%) | | 22/77 (29%) | 6/15 (40%) | |
| Grade 2 | 83/231 (36%) | 63/160 (39%) | | 24/77 (31%) | 8/15 (53%) | |
| Grade 3 | 52/231 (23%) | 23/160 (14%) | | 29/77 (38%) | 1/15 (7%) | |
| **Time from stroke onset to door of first hospital, minutes – median (IQR)** | 67 (45-125) | 62 (40-111) | 0.119 | 364 (295-517) | 316 (295-371) | 0.178 |

ASPECTS = Alberta Stroke Program Early CT Score; CTA = computed tomography angiography; ICA = intracranial part of internal carotid artery; IQR = interquartile range; IVT = intravenous thrombolysis; M1 = first segment of the middle cerebral artery; M2 = second segment (after first bifurcation) of the middle cerebral artery; mRS = modified Rankin Scale; NCCT = non-contrast computed tomography; NIHSS = National Institutes of Health Stroke Scale; no. = number; SD = standard deviation.

Number of missing values: [a]8; [b]10; [c]15; [d]9; [e]75.
