# Supplementary material for: Interhospital transfer vs. direct presentation of patients with a large vessel occlusion not eligible for IV thrombolysis
Source: J Neurol. 2020 Apr 7;267(7):2142–50. doi: 10.1007/s00415-020-09812-5 (PMC7320925; doi:10.1007/s00415-020-09812-5)
Supplement: Supplementary file 3 — Supplementary file3 (PDF 158 kb) [file 415_2020_9812_MOESM3_ESM.pdf]

ELECTRONIC SUPPLEMENTAL MATERIAL: ONLINE RESOURCE 3

TRANSFER VS. DIRECT PRESENTATION TO A COMPREHENSIVE STROKE CENTER OF PATIENTS WITH A LARGE VESSEL OCCLUSION NOT ELIGIBLE FOR IV THROMBOLYSIS

Laura C.C. van Meenen[1], Adrien E. Groot[1], Esmee Venema[2], Bart J. Emmer[3], Martin D. Smeekes[4], Geert Jan Kommer[5], Charles B.L.M. Majoie[3], Yvo B.W.E.M. Roos[1], Wouter J. Schonewille[6], Bob Roozenbeek[7], Jonathan M. Coutinho[1] on behalf of the MR CLEAN Registry Investigators[8]

1. Department of Neurology, Amsterdam University Medical Centers, University of Amsterdam, the Netherlands
2. Department of Neurology and Department of Public Health, Erasmus MC University Medical Center, Rotterdam, the Netherlands
3. Department of Radiology and Nuclear Medicine, Amsterdam University Medical Centers, University of Amsterdam, the Netherlands
4. Emergency Medical Services North-Holland North, Alkmaar, the Netherlands
5. National Institute of Public Health and the Environment, Center for Nutrition, Prevention and Health services
6. Department of Neurology, St. Antonius Ziekenhuis, Nieuwegein, the Netherlands
7. Department of Radiology & Nuclear Medicine, Erasmus MC University Medical Center, Rotterdam, the Netherlands
8. MR CLEAN Registry Investigators - group authors: please see Online Resource 1

Corresponding author: dr. J.M. Coutinho (telephone: +31 20 732 2289, email: j.coutinho@amsterdamumc.nl)

Online Resource 3. Treatment times stratified by presentation within 4.5 hour time window

	**Presentation ≤ 4.5 hours**				**Presentation > 4.5 hours**			
	**Direct, n=290**	**Transfer, n=186**	**Unadjusted β (95% CI)**	**Adjusted β (95% CI)**	**Direct, n=90**	**Transfer, n=18**	**Unadjusted β (95% CI)**	**Adjusted β (95% CI)**
**Onset-to-groin time[a] – median (IQR)**	180 (129-255)	228 (180-310)	**-38.3 (-56.4 to -20.1)**	**-46.4 (-66.1 to -26.6)[b]**	469 (370-625)	455 (415-590)	7.19 (-91.4 to 105.6)	-8.1 (-115.9 to 99.7)[b]
**Travel time-corrected onset-to-groin time[c] – median (IQR)**	180 (129-255)	208 (156-279)	-16.3 (-34.4 to 1.7)	**-23.7 (-43.3 to -4.1)[b]**	469 (370-625)	436 (373-603)	33.6 (-65.1 to 132.3)	19.7 (-88.4 to 127.7)[b]
**First-door-to-groin time[a] – median (IQR)**	94 (73-125)	150 (116-186)	**-47.6 (-61.0 to -34.3)**	**-50.8 (-65.7 to -36.2)[b]**	84 (69-114)	139 (100-191)	**-56.7 (-90.0 to -23.5)**	**-47.0 (-71.7 to -22.3)[b]**
**Travel time-corrected first-door-to-groin time[c] – median (IQR)**	94 (73-125)	128 (97-166)	**-25.7 (-39.0 to -12.5)**	**-28.3 (-43.3 to -13.3)[b]**	84 (69-114)	117 (77-168)	-30.3 (-63.5 to 2.8)	-19.2 (-43.7 to 5.3)[b]

CI = confidence interval; IQR = interquartile range.

[b]Adjusted for age, pre-stroke mRS, baseline blood pressure, baseline NIHSS and location of occlusion.

Number of missing values: [a]5; [c]10.
